# Sleep–Wake Disturbances in Patients With Chronic Pain—Associations With Physical Activity Levels

**DOI:** 10.1002/ejp.70330

**Published:** 2026-07-06

**Authors:** Emma Varkey, Martin Olsson, Adam Piasecki, Jan Hedner, Ludger Grote, Mats Börjesson, Daniel Arvidsson, Paulin Andréll

**Affiliations:** ^1^ Department of Occupational Therapy and Physical Therapy Sahlgrenska University Hospital/Östra Gothenburg Region Västra Götaland Sweden; ^2^ Department of Health and Rehabilitation/Physiotherapy Institute of Neuroscience and Physiology, Sahlgrenska Academy, University of Gothenburg Gothenburg Sweden; ^3^ Department of Anaesthesiology and Intensive Care Medicine Institute of Clinical Sciences, Sahlgrenska Academy, University of Gothenburg Gothenburg Sweden; ^4^ Department of Anaesthesiology, Intensive Care Medicine and Pain Medicine Sahlgrenska University Hospital/Östra Gothenburg Region Västra Götaland Sweden; ^5^ Centre for Sleep and Wake Disorders Institute of Medicine, Sahlgrenska Academy, University of Gothenburg Gothenburg Sweden; ^6^ Department of Anaesthesiology and Intensive Care Medicine Sahlgrenska University Hospital Mölndal Region Västra Götaland Sweden; ^7^ Sleep Disorders Centre, Pulmonary Department Sahlgrenska University Hospital Gothenburg Region Västra Götaland Sweden; ^8^ Center for Lifestyle Intervention, Department of Medicine, Geriatrics and Emergency Care Sahlgrenska University Hospital/University of Gothenburg Gothenburg Sweden; ^9^ Department of Molecular and Clinical Medicine Institute of Medicine, Sahlgrenska Academy, University of Gothenburg Gothenburg Sweden; ^10^ Center for Health and Performance, Department of Food and Nutrition, and Sport Science University of Gothenburg Gothenburg Sweden

## Abstract

**Background:**

Sleep–wake disturbances are frequently reported by patients with chronic pain. Physical activity, recommended as a first‐line treatment for chronic pain, can be difficult to implement due to pain severity. This study aimed to assess the prevalence of insomnia and other sleep–wake disturbances in a cohort of patients with chronic pain and to explore the influence of physical activity, opioid use, reported pain, and symptoms of depression and anxiety.

**Methods:**

This clinical cross‐sectional study included 100 patients with chronic pain attending a specialist‐level pain clinic. Participants were consecutively enrolled, and sleep and physical activity were evaluated using accelerometers. Additional assessments included the Insomnia Severity Index (ISI), Pittsburgh Sleep Quality Index (PSQI), Epworth Sleepiness Scale (ESS), symptoms of restless legs syndrome (RLS), STOP‐Bang score, pain intensity assessed by Numeric Rating Scale (NRS), Hospital Anxiety and Depression Scale (HADS), and self‐reported level of physical activity.

**Results:**

Overall, nearly all participants experienced sleep–wake disturbances. Average sleep duration (hh:mm) was 5:30 (range, 1:46–9:52), and sleep efficiency was 64% (range, 28–81). Based on PSQI scores, 95% were classified as having poor sleep quality. Clinical insomnia (ISI ≥ 15p) was reported by 67%. A statistically significant correlation (*r*_s_ = 0.31, 95% CI, 0.10–0.50) was found between sleep efficiency and level of physical activity and between ISI and HADS depression (*r*_s_ = 0.41, 95% CI, 0.22–0.57).

**Conclusions:**

A wide range of sleep–wake disturbances were present in the examined cohort, highlighting the possible benefit of assessing sleep to optimise pain rehabilitation.

**Significance Statement:**

In this cohort, nearly all patients with chronic pain experienced sleep–wake disturbances, and none reached 85% sleep efficiency indicating stable sleep. These findings emphasise the need for a comprehensive assessment of sleep problems to improve the understanding and management of severe pain.

**Trial Registration:**

ClinicalTrials.gov: NCT04649281

## 1 Background

Chronic pain affects approximately 20% of adults in Europe (Breivik et al. 2006) and is associated with reduced social and physical functioning (Hadi et al. 2019; Jackson et al. 2014), impaired mental health, anxiety, depression, fatigue, and sleep disorders (Aaron et al. 2025; Finan et al. 2013; Smith and Haythornthwaite 2004). An estimated 40%–88% of patients with chronic pain report sleep–wake disturbances (Jain et al. 2024), manifesting as disruptions in sleep quality, quantity, or timing, as well as increased daytime sleepiness and fatigue (Grandner 2014). Common comorbidities in pain conditions, such as obstructive sleep apnea (OSA) (Larsen et al. 2022) and restless legs syndrome (RLS), can further contribute to sleep disturbances (Stehlik et al. 2014).

There is a bilateral connection between pain and sleep–wake disturbances. Disturbed sleep is an important modulator and causes perceived pain (Finan et al. 2013; Smith and Haythornthwaite 2004). Longitudinal studies have shown that sleep–wake disturbances reliably predict the incidence and aggravation of chronic pain (Jansson‐Frojmark and Boersma 2012; Skarpsno et al. 2019). Further, impaired sleep is suggested as a stronger and more reliable predictor of pain than pain is of sleep impairment (Runge et al. 2024). Given the pain‐reducing role of sleep, identifying and treating sleep–wake disturbances may yield positive pain‐modulating effects in chronic pain conditions (Finan et al. 2013).

Despite a lack of evidence for long‐term treatment, 5%–7% of patients with chronic pain regularly use strong opioids (Busse et al. 2018; Chou et al. 2015; Ekholm et al. 2014); this proportion exceeds 20% among patients with chronic non‐cancer pain (Dahlhamer et al. 2021; Grelz et al. 2022). Current data suggest that opioids disrupt one or more components of sleep (Cutrufello et al. 2020). These detrimental effects on sleep quality may negatively impact overall disease management in patients with chronic pain (Cao and Javaheri 2018; Frers et al. 2021).

Physical activity is recommended as a primary intervention for patients with chronic pain (Vaegter et al. 2024). There is an association between higher levels of physical activity and lower chronic pain prevalence (inverse dose–response) (Fjeld et al. 2023) and a positive association between physical activity and sleep quality (Yang et al. 2012; Zou et al. 2019). Nevertheless, patients with chronic pain may have difficulties meeting the WHO recommendations of > 150 min/week of moderate‐to‐vigorous physical activity (MVPA) (Varkey et al. 2022).

To our knowledge, the relationships between sleep–wake disturbances, level of physical activity, and opioid use have not been studied simultaneously. To increase the understanding of sleep–wake disturbances in chronic pain, this study aimed: (1) to assess the prevalence of insomnia and other sleep–wake disturbances in a cohort of patients with chronic pain; (2) to explore potential correlations with physical activity levels and opioid use; and (3) to examine relationships between sleep–wake disturbances and pain outcomes, anxiety, and depression.

## 2 Methods

### 2.1 Participants

This cross‐sectional study included 100 patients referred to the Pain Centre of Sahlgrenska University Hospital in Gothenburg, Sweden, who were consecutively recruited to the study between November 2020 and May 2024.

Inclusion criteria for the study were: chronic pain (i.e., pain > 3 months), planned follow‐up at the Pain Centre, age ≥ 18 years, and consent to participate in the study. Exclusion criteria were: inadequate communication skills in the Swedish language, alcohol or substance abuse, severe, untreated psychiatric disorders, including psychiatric disease and/or psychological conditions that were the primary determinant of the patient's pain condition, and malignant disease with expected short‐term survival.

### 2.2 Data Collection

#### 2.2.1 Sleep

Participants were screened for sleep–wake disturbances using several validated questionnaires. The Insomnia Severity Index (ISI) addresses insomnia symptoms and consists of 7 questions rated on a Likert scale (scoring range 0–28); a score of ≥ 15 is considered to indicate moderate insomnia and > 21 severe insomnia (Dragioti et al. 2015; Morin et al. 2011). Accordingly, an ISI score of ≥ 15 is generally interpreted as clinically significant insomnia. The Pittsburgh Sleep Quality Index (PSQI) consists of 10 questions divided into subdomains and generates a global sleep quality score (scoring range 0–21), where a score of > 5 points indicates the presence of a sleep disorder (Buysse et al. 1989). The Epworth Sleepiness Scale (ESS) consists of 8 questions and quantifies daytime sleepiness (scoring range 0–24); a score of > 9 signifies excessive daytime sleepiness (Kendzerska et al. 2014). Participants were also screened for OSA with the STOP‐Bang questionnaire (scoring range 0–8) to assign the level of risk as low (0–2), intermediate (3–4), and high (5–8) (Nagappa et al. 2015), and for RLS with a symptoms screening questionnaire (scoring range 0–4) (Walters 1995). Finally, the participants answered a question regarding their chronotype (‘To what extent do you consider yourself a morning or evening person?’) to elucidate morning, intermediate, or evening type.

Participants wore one accelerometer (GENEActiv Original) for sleep analysis, placed on the non‐dominant wrist for one week. A validated Sadeh algorithm was used to analyse sleep–wake data (Kosmadopoulos et al. 2014; Sadeh et al. 1994), including sleep duration and sleep efficiency (sleep duration/total time in bed). Regarding sleep duration, there is no universally accepted cut‐off for what constitutes ‘healthy’ sleep. However, a consensus statement from the American Academy of Sleep Medicine states that adults are recommended to sleep > 7 h per night on a regular basis and that 7–9 h of sleep are appropriate to support optimal health (Watson et al. 2015); this definition was used in this study. A sleep efficiency of ≥ 85% is described as an appropriate indicator of good sleep quality, while < 75% is indicative of poor sleep quality (Ohayon et al. 2017). The participants also filled out a subjective sleep diary regarding estimated sleep duration during the same week.

#### 2.2.2 Physical Activity

The level of physical activity was determined from triaxial accelerometer data collected at the right hip with the Axivity AX3 (Axivity Ltd., Newcastle upon Tyne, UK), worn in an elastic belt around the waist for one week. Raw accelerometer data were processed to a measure of physical activity intensity (milligravity, mg) in 3‐s epochs using the 10 Hz Frequency Extended Method (Fridolfsson et al. 2018; Fridolfsson et al. 2019), and thereafter to time spent in MVPA using a lab‐based calibration equation relating mg to a reference measure of physical activity intensity from measured oxygen uptake (Metabolic Equivalent of Task, MET) (Fridolfsson et al. 2019). Commonly, 3 METs is used as a cut‐off point for MVPA (Migueles et al. 2017). However, this cut‐off point has been shown to be too low for most individuals in the general population in relation to the association with cardiometabolic health (Fridolfsson et al. 2023). Thus, to define MVPA in the present study, a more appropriate cut‐off point that promotes cardiorespiratory fitness, corresponding to 4.5 METs (Fridolfsson et al. 2024), was used. Non‐wear time was defined as 60 min of zero acceleration with an allowance of up to 2 min of interruptions (Troiano et al. 2008), which was excluded from the data to be analysed. A valid day was defined as at least 10 h of wear‐time (Migueles et al. 2017). Questionnaire data regarding self‐reported levels of physical activity were obtained from the Swedish Quality Registry for Pain Rehabilitation (SQRP) and expressed as minutes per week of MVPA (moderate‐to‐vigorous physical activity), calculated as MPA (moderate physical activity) + 2 × VPA (vigorous physical activity) (Lönn et al. 2022).

#### 2.2.3 Pain‐Associated Comorbidities and Patient Health Characteristics

Patient health data were obtained from the SQRP, and data from the registry were collected before the first physician visit at the Pain Centre. The registry also provided data collected using validated questionnaires, including the Hospital Anxiety and Depression Scale (HADS) (Zigmond and Snaith 1983) and questions about physical activity (Lönn et al. 2022; Olsson et al. 2016). Self‐reported data were used to determine body mass index (BMI), pain duration, and number of pain locations. Participants reported the presence of pain (yes/no) in 36 predefined anatomical regions. A total pain distribution score was calculated by summing the number of regions in which pain was reported, yielding a possible range of 0–36. Participants who had not completed these questionnaires in the SQRP before inclusion were offered the opportunity to do so. Those who declined or failed to do so were excluded from the study. In addition to the registry questionnaires, the participants reported current pharmacological treatments, including opioids, and their doses. The participants completed the questionnaires once during the study period. They were also asked to document their daily pain during rest and movement according to the Numeric Rating Scale (NRS), using a pain diary over one week, in conjunction with the sleep diary. Pain diagnoses were obtained from medical records from the first physician visit at the Pain Centre. All data except for the SQRP were collected during the same week. For accelerometer data as well as sleep diaries, patients with fewer than 4 out of 7 valid days were excluded from the analyses.

### 2.3 Statistical Methods

The sample size was calculated based on a previous analysis of SQRP data for patients at the Pain Centre, in which the primary outcome was insomnia, defined according to the ISI. This indicated that 100 patients would be sufficient to ensure variability in this outcome, and no subgroup (according to the ISI scores) was expected to be smaller than *n* = 20.

SPSS version 28.0, R version 4.4.2 and RStudio version 2025.05.0 + 496 were used for the statistical analyses. Spearman's correlation test was performed to determine the correlation between ranked variables. Partial correlations were estimated while controlling for specified covariates (gender, age, and BMI). Because the regression models used to obtain residuals for partial correlation require complete data, multiple imputation by chained equations with 20 imputed datasets was applied, using the mice package (version 3.17.0) (van Buuren 2011). Variable‐specific imputation methods were used: classification and regression trees for continuous variables and multinomial logistic regression for categorical variables. For each of the imputed datasets, the predictor and outcome variables were separately regressed on the control variables, and residuals were extracted (Waliczek 1996). The Spearman correlation (*ρ*) between these residuals represented the association between the predictor and outcome after removing the linear effects of the controls. The post‐imputation process requires pooling results across the imputed datasets (Austin et al. 2021; Heymans and Eekhout 2019), which was performed using Rubin's rules (Rubin 1987; Austin et al. 2021; Heymans and Eekhout 2019). Correlation coefficients, bounded between −1 and 1, follow a uniform distribution, whereas Rubin's rules assume approximately normally distributed estimates; therefore, they were transformed to Fisher's *z* scale. Standard errors were calculated using the approximation method for Fisher's *z*‐transformed partial correlations, accounting for the number of control variables. Pooled point estimates on the *z* scale were obtained using Rubin's rules, combining the average within‐imputation variance and the between‐imputation variance to derive the total variance, from which pooled standard errors, *t*‐statistics, and two‐sided *p*‐values were computed. Final results are reported as pooled partial correlations with 95% confidence intervals (95% CIs; back‐transformed to the original correlation scale). Differences between groups were calculated using ANCOVA, adjusted for age, gender, and BMI, and data are presented as mean differences with 95% CIs. All significance tests were two‐sided and conducted at the 5% significance level. Continuous variables are described using mean (SD), and categorical variables are reported as numbers and valid percentages.

### 2.4 Ethics

The study was approved by the Swedish Ethical Review Authority for Medical Research (registration number 2019‐05496, supplementary applications registration numbers 2020–03959; 2023‐01978‐02) and conducted in accordance with the Declaration of Helsinki. All participants received oral and written study information, and oral and written informed consent was obtained from all study participants prior to enrollment.

## 3 Results

### 3.1 Participants

**FIGURE 1:**
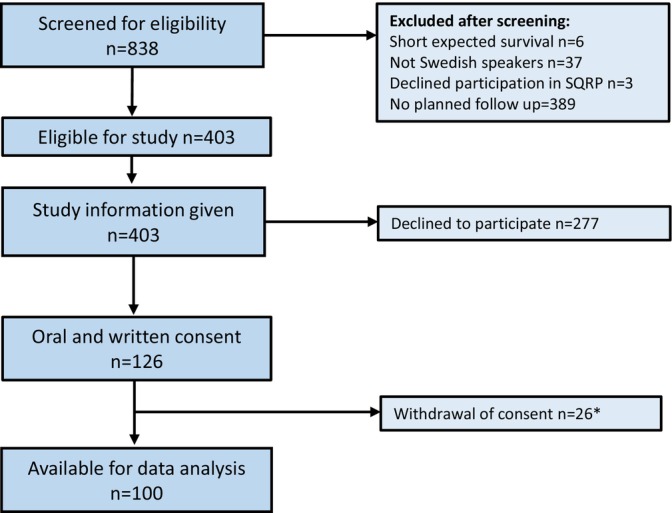
Flowchart of the study participants. *No return of data were considered withdrawal of consent. Six participants communicated a withdrawal of consent to participate in the study and 20 declined to provide data. SQRP, Swedish Quality Registry for Pain Rehabilitation.

**FIGURE 2:**
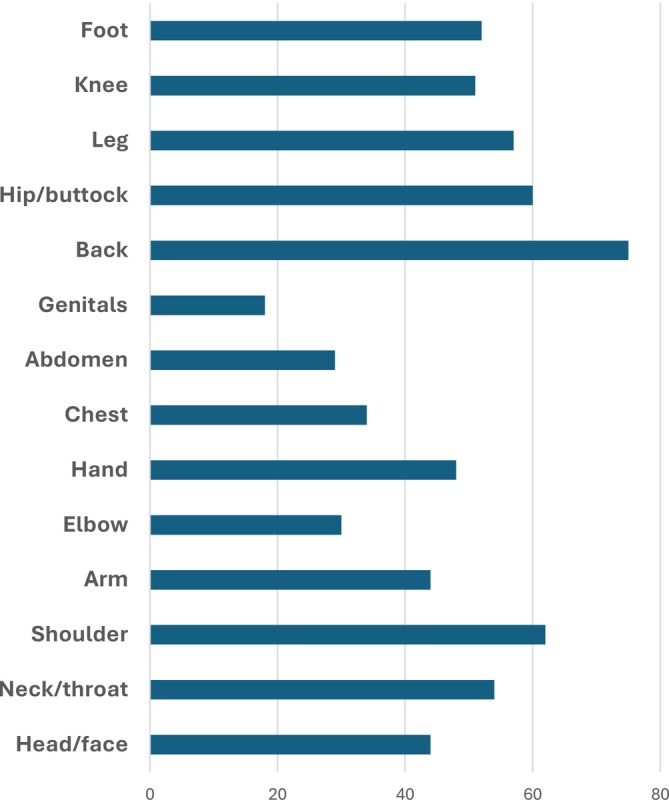
Pain locations reported by the participants (*n* = 100).

A total of 100 patients with chronic pain participated in the study (see the study flowchart in Figure 1). Gender was reported by all but one participant (62 women and 37 men). The participants had nociceptive (28%), neuropathic (19%), and nociplastic (32%) pain as their primary diagnosis. Six % had pain of unknown origin, and 15% were not diagnosed according to a pain mechanism. The most common pain locations were back (*n* = 75), followed by shoulder (*n* = 62) and hip/buttocks (*n* = 60). The pain locations are described in Figure 2, and demographic data for the study population are presented in Table 1.

**TABLE 1:** Baseline demographic and clinical characteristics for the total study population (*n* = 100) and by gender (women *n* = 62 and men *n* = 37).

	All participants (*n* = 100)^a^	Women (*n* = 62)	Men (*n* = 37)
Age^A^	47 (13)	47 (13)	48 (13)
BMI (kg/m^2^)^A^	28 (7)	27 (7)	30 (6)
Pain			
*Primary pain diagnosis* ^B^			
Nociceptive pain	28 (28)	19 (31)	9 (24)
Neuropathic pain	32 (32)	18 (29)	14 (38)
Nociplastic pain	19 (19)	16 (26)	2 (5)
Pain of unknown origin	6 (6)	2 (3)	4 (11)
Not classified according to pain mechanism	15 (15)	7 (11)	8 (22)
Pain duration, (years)^B^	8 (8)	8 (8)	9 (9)
< 1 year	7 (8)	6 (10)	1 (3)
1–5 years	41 (45)	24 (41)	17 (52)
6–10 years	15 (17)	9 (16)	6 (18)
> 10 years	28 (31)	19 (33)	9 (27)
Pain locations, *n* (0–36)^A^	14 (9)	15 (10)	12 (8)
Education, *n (%)* ^B^			
Primary school	11 (12)	7 (12)	4 (12)
High school	42 (46)	24 (41)	18 (55)
University	37 (40)	27 (46)	10 (30)
Other	2 (2)	1 (2)	1 (3)
Currently working, *n (%)* ^B^	46 (70)	26 (65)	20 (77)

*Note:* Data are reported as ^A^mean (SD) or ^B^number (valid %).

^a^ One participant did not select either ‘male’ or ‘female’ when asked about gender.

### 3.2 Prevalence of Sleep Disturbances

**FIGURE 3:**
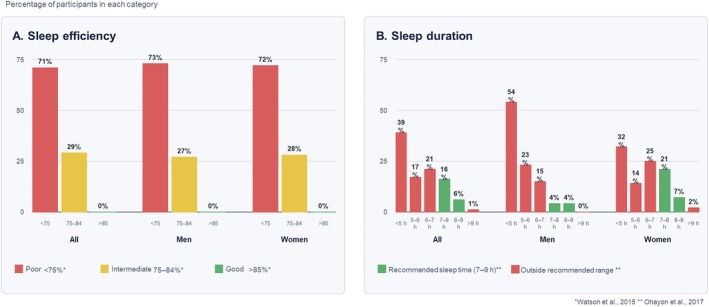
(a) Sleep efficiency and (b) sleep duration reported by gender, categorised according to recommended thresholds.

The participants reported symptoms associated with several sleep–wake disturbances and presented objective signs of sleep disturbances on accelerometry. They had a mean accelerometer‐measured sleep duration of 5 h and 30 min (range, 1 h 46 min–9 h 52 min) and a sleep efficiency of 64% (range, 28–81). Nearly 80% of participants did not meet the recommendations of 7–9 h of sleep per night, and none reached > 85% sleep efficiency (Figure 3).

Symptoms consistent with clinical insomnia, defined as an ISI score of 15–28, was reported by 67% of the participants, and symptoms consistent with severe insomnia, defined as an ISI score of 22–28, was reported by almost 30%. Only 10% (*n* = 9) of the participants reported no insomnia (ISI score of 0–7). Most of the participants, 95%, scored > 5 points on the PSQI, indicating poor sleep quality and suggesting the presence of sleep disturbances. About 60% reported symptoms of RLS, while 40% reported excessive daytime sleepiness, with an EES score of > 9. A high risk of OSA, defined as a STOP‐Bang score of ≥ 5, was reported by 17% of the participants. The characteristics of sleep, physical activity, pain, and symptoms of anxiety and depression for the total study population and by gender are presented in Table 2.

**TABLE 2:** Sleep, pain, physical activity, and symptoms of anxiety and depression for the total study population (*n* = 100) and by gender (women *n* = 62 and men *n* = 37).

	All patients^a^ (*n* = 100)	Women (*n* = 62)	Men (*n* = 37)	Mean diff (95% CI) (women–men)^b^
**Sleep**				
*Accelerometer* ^c^				
*7‐day average*				
Sleep duration (hh:min)	5:30 (1:47)	5:45 (1:55)	5:01 (1:27)	36 (−23 to 95)^d^
Sleep efficiency *(%, sleep duration/total time in bed)*	64 (14)	64 (15)	63 (12)	−1.2 (−8.2 to 5.9)
*Diary, 7‐day average* ^e^				
Estimated sleep duration	6:32 (1:16)	6:42 (1:23)	6:07 (0:51)	37 (−3.6 to 78.2)^d^
*Chronotype*				
Early chronotype	24 (27)	18 (31)	6 (20)	
Late chronotype	39 (27)	22 (37)	16 (53)	
Neither	24 (43)	16 (27)	8 (27)	
Do not know	3 (3)	3 (5)	0 (0)	
*ISI score (0–28p)* ^f^	16.8 (6.9)	16.1 (7.4)	18.2 (5.8)	−1.8 (−5.1 to 1.4)
0–14 no to mild insomnia	31 (33)	24 (40)	7 (21)	
15–21 moderate insomnia	37 (39)	19 (32)	18 (53)	
22–28 severe insomnia	26 (28)	17 (28)	9 (27)	
*ESS score (0–24p)*	8.5 (5.4)	8.7 (5.7)	8.0 (5.1)	0.6 (−2.0 to 3.2)
0–9 normal	55 (60)	33 (56)	22 (69)	
> 9 excessive daytime sleepiness	37 (40)	26 (44)	10 (31)	
*PSQI score (0–21p)* ^g^	11.9 (4.1)	11.6 (4.3)	12.6 (3.7)	−0.1 (−2.0 to 1.9)
0–5 good sleep quality	4 (5)	3 (6)	1 (4)	
> 5 poor sleep quality	76 (95)	49 (94)	26 (96)	
*RLS symptoms (0–4p)* ^h^	1.8 (1.6)	1.8 (1.6)	1.8 (1.7)	−0.2 (−1.0 to 0.6)
0	36 (39)	23 (39)	12 (38)	
1	5 (5)	2 (3)	3 (9)	
2	16 (17)	13 (22)	3 (9)	
3	14 (15)	8 (14)	6 (19)	
4	21 (23)	13 (22)	8 (25)	
*STOP‐Bang score (0‐8p)*	2.7 (1.8)	1.8 (1.1)	4.3 (1.8)	−1.9 (−2.4 to −1.4)**
0–2 low risk of sleep apnea	51 (55)	42 (71)	8 (25)	
2–4 intermediate risk of sleep apnea	25 (27)	16 (27)	9 (28)	
> 4 high risk of sleep apnea	16 (17)	1 (2)	15 (47)	
**Pain** ^i^				
*NRS (0–10)*	5.9 (1.9)	6.1 (1.9)	5.5 (1.9)	0.4 (−0.5 to 1.4)
*7‐day average rest*				
*NRS (0–10)*	6.4 (2.0)	6.5 (2.0)	6.0 (2.0)	0.6 (−0.4 to 1.6)
*7‐day average motion*				
**Physical activity**				
*Accelerometer* ^j^				
*MVPA min/week*	40 (48)	43 (52)	36 (40)	−4 (−28 to 20)
*Self‐reported* ^k^				
*MVPA min/week*	212 (162)	222 (162)	197 (164)	0 (−71 to 71)
**Anxiety and depression** ^l^				
*HADS Anxiety (0–21p)*	8.8 (5.1)	8.9 (5.2)	8.7 (5.0)	0.6 (−1.7 to 2.9)
*HADS Depression (0–21p)*	9.1 (4.7)	8.6 (4.8)	9.8 (4.5)	−0.7 (−2.8 to 1.5)

*Note:* Data are reported as mean (SD). Groups of participants are reported as number (valid %).

Abbreviations: ESS, Epworth Sleepiness Scale (measures daytime sleepiness); HADS, Hospital Anxiety and Depression Scale (measures symptoms of anxiety and depression); ISI, Insomnia Severity Index (assesses the severity of insomnia); MVPA, moderate‐to‐vigorous physical activity (measures physical activity levels); NRS, numeric rating scale (used for pain intensity assessment); PSQI, Pittsburgh Sleep Quality Index (evaluates overall sleep quality); RLS, restless legs syndrome (a neurological condition causing an urge to move the legs).

^a^ One participant did not select either ‘male’ or ‘female’ when asked about gender.

^b^ Mean difference (95% CI) adjusted for age and BMI.

^c^ *n* = 70.

^d^ Mean difference reported in minutes.

^e^ *n* = 73.

^f^ *n* = 94.

^g^ *n* = 80.

^h^ Number of cardinal symptoms (0–4).

^i^ *n* = 86.

^j^ *n* = 84.

^k^ *n* = 98.

^l^ *n* = 98.

** *p* < 0.001.

A group comparison showed that participants reporting symptoms corresponding to clinical significant insomnia according to ISI had higher levels of depression symptoms as measured by HADS than those reporting no or mild insomnia (Table 3). No differences were found between the groups regarding sleep–wake disturbances, pain, or level of physical activity.

**TABLE 3:** Sleep, pain, physical activity, and symptoms of anxiety and depression in participants who reported symptoms of clinical insomnia (moderate or severe insomnia, ISI score of 15–28) and those who reported no or mild insomnia (0–14).

	No or mild insomnia (*n* = 31)	Moderate or severe insomnia (*n* = 63)	Mean difference (95% CI)^a^ (No or mild–Moderate or severe)
Age (years)	46 (15)	47 (12)	
Gender, women/men, *n*	24/7	36/27	
BMI (kg/m^2^)	27.0 (6.1)	28.7 (6.8)	
**Sleep**			
*Accelerometer*			
Sleep duration (hh:min)	5:43 (1:52)	5:22 (1:43)	−6.0 (−69.6 to 57.5)^b^
Sleep efficiency *(%, sleep duration/total time in bed)*	66 (13)	63 (14)	−1.2 (−8.9 to 6.5)
*Diary, 7‐day average*			
Estimated sleep duration (hh:min)	7:01 (0:45)	6:13 (1:27)	36.4 (−3.1 to 76.0)
*ESS score (0–24p)*	8.6 (5.1)	8.4 (5.9)	−0.7 (−3.5 to 2.1)
0–9 normal	19 (61)	33 (59)	
> 9 excessive day‐time sleepiness	12 (39)	23 (41)	
*PSQI score (0–21p)*	8.3 (3.5)	13.9 (2.9)	−4.8 (−6.4 to −3.2)***
0–5 good sleep quality	4 (15)	0 (0)	
> 5 poor sleep quality	23 (85)	50 (100)	
*RLS symptoms (0–4)* ^c^	1.5 (1.5)	1.9 (1.7)	−0.2 (−1.0 to 0.6)
0	14 (45)	20 (36)	
1	1 (3)	4 (7)	
2	6 (19)	10 (18)	
3	7 (23)	7 (13)	
4	3 (10)	15 (27)	
*STOP‐Bang score (0–8p)*	2.3 (1.8)	2.8 (1.8)	0.18 (−0.4 to 0.7)
0–2 low risk of sleep apnea	18 (60)	30 (53)	
2–4 intermediate risk of sleep apnea	9 (30)	16 (28)	
> 4 high risk of sleep apnea	3 (10)	11 (19)	
**Pain**			
*NRS (0–10p), 7‐day average rest*	5.3 (2.2)	6.3 (1.6)	−0.9 (−1.8 to 0.0)
*NRS (0–10p), 7‐day average motion*	6.4 (2.2)	6.3 (1.8)	−0.2 (−1.1 to 0.8)
Pain locations (0–36)	12 (8)	15 (10)	−3.8 (−8.1 to 0.6)
**Physical activity**			
*Accelerometer, MVPA min/week*	50 (56)	37 (44)	9 (−16 to 34)
*Self‐reported*	253 (147)	187 (163)	55 (−20 to 131)
*MVPA min/week*			
**Anxiety and depression**			
*HADS Anxiety (0–21p)*	7.8 (5.1)	9.3 (4.9)	−2.3 (−4.6 to 0.1)
*HADS Depression (0–21p)*	7.0 (4.7)	10.1 (4.1)	−3.6 (−5.7 to −1.5)***

*Note:* Data are reported as mean (SD) or number (%).

Abbreviations: ESS, Epworth Sleepiness Scale (measures daytime sleepiness); HADS, Hospital Anxiety and Depression Scale (measures symptoms of anxiety and depression); ISI, Insomnia Severity Index (assesses the severity of insomnia); MVPA, Moderate‐to‐Vigorous Physical Activity (measures physical activity levels); NRS, Numeric Rating Scale (used for pain intensity assessment); PSQI, Pittsburgh Sleep Quality Index (evaluates overall sleep quality); RLS, Restless Legs Syndrome (a neurological condition causing an urge to move the legs).

^a^ Adjusted for BMI, gender, and age.

^b^ Mean difference reported in minutes.

^c^ Number of cardinal symptoms (0–4).

*** *p* < 0.001.

### 3.3 Correlations Between Sleep Disturbances and Physical Activity

**FIGURE 4:**
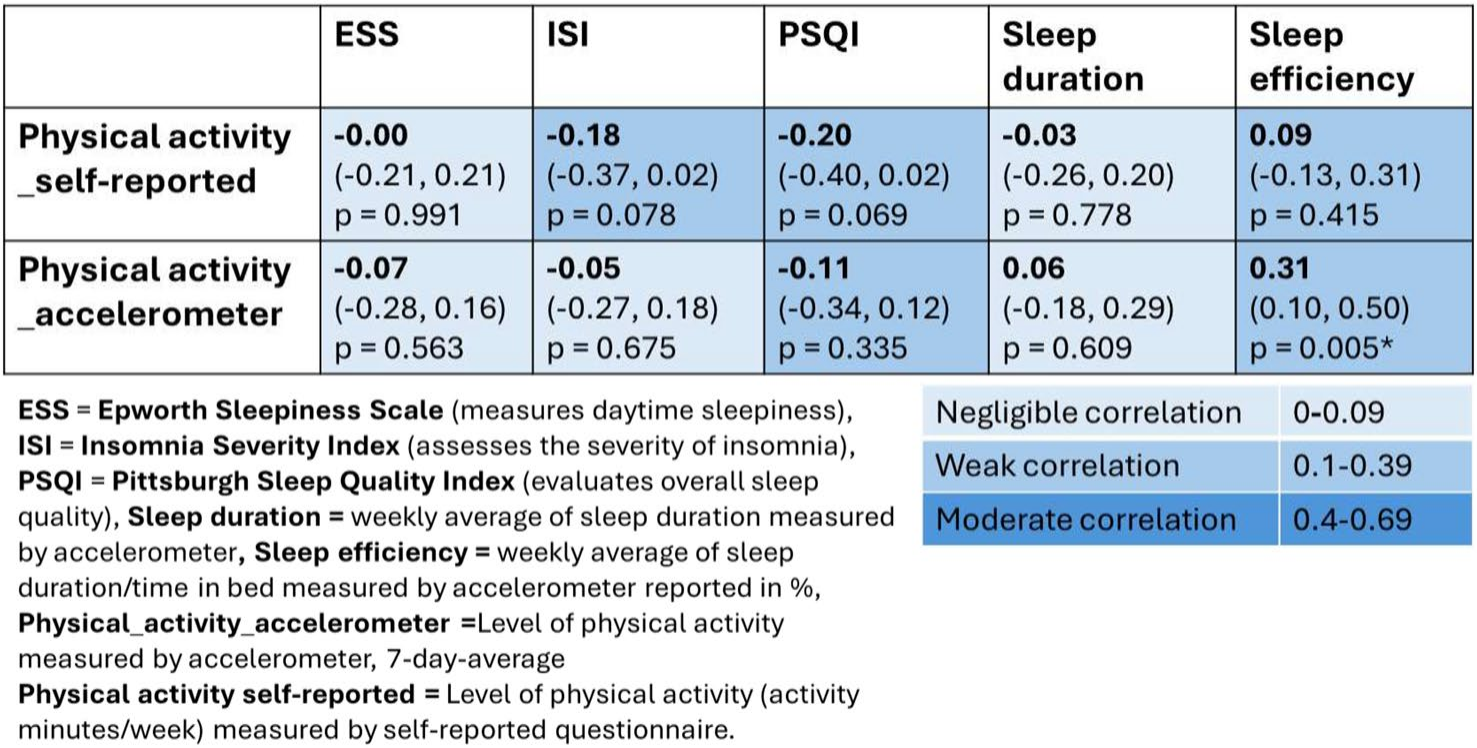
Spearman's correlations between sleep–wake disturbances and level of physical activity. *Significant at the 0.05‐level.

There was a weak positive correlation between the accelerometer‐measured level of physical activity and sleep efficiency (*r*_s_ = 0.31, 95% CI, 0.10–0.50). No other significant correlations were found between sleep disturbances and level of physical activity (Figure 4).

### 3.4 Differences Between Opioid Users and Non‐Opioid Users

One third of the participants reported opioid use (oxycodone, *n* = 15; tapentadol, *n* = 13; buprenorphine, *n* = 5; codeine, *n* = 3; methadone, *n* = 2; fentanyl, *n* = 1). There were no differences between users and non‐users of opioids regarding sleep disturbances except for the STOP‐Bang score, where participants using opioids had a higher risk of OSA. No differences were observed with respect to pain, level of physical activity, or symptoms of anxiety or depression (Table S1).

### 3.5 Correlations Between Sleep Disturbances and Pain, Anxiety, and Depression

**FIGURE 5:**
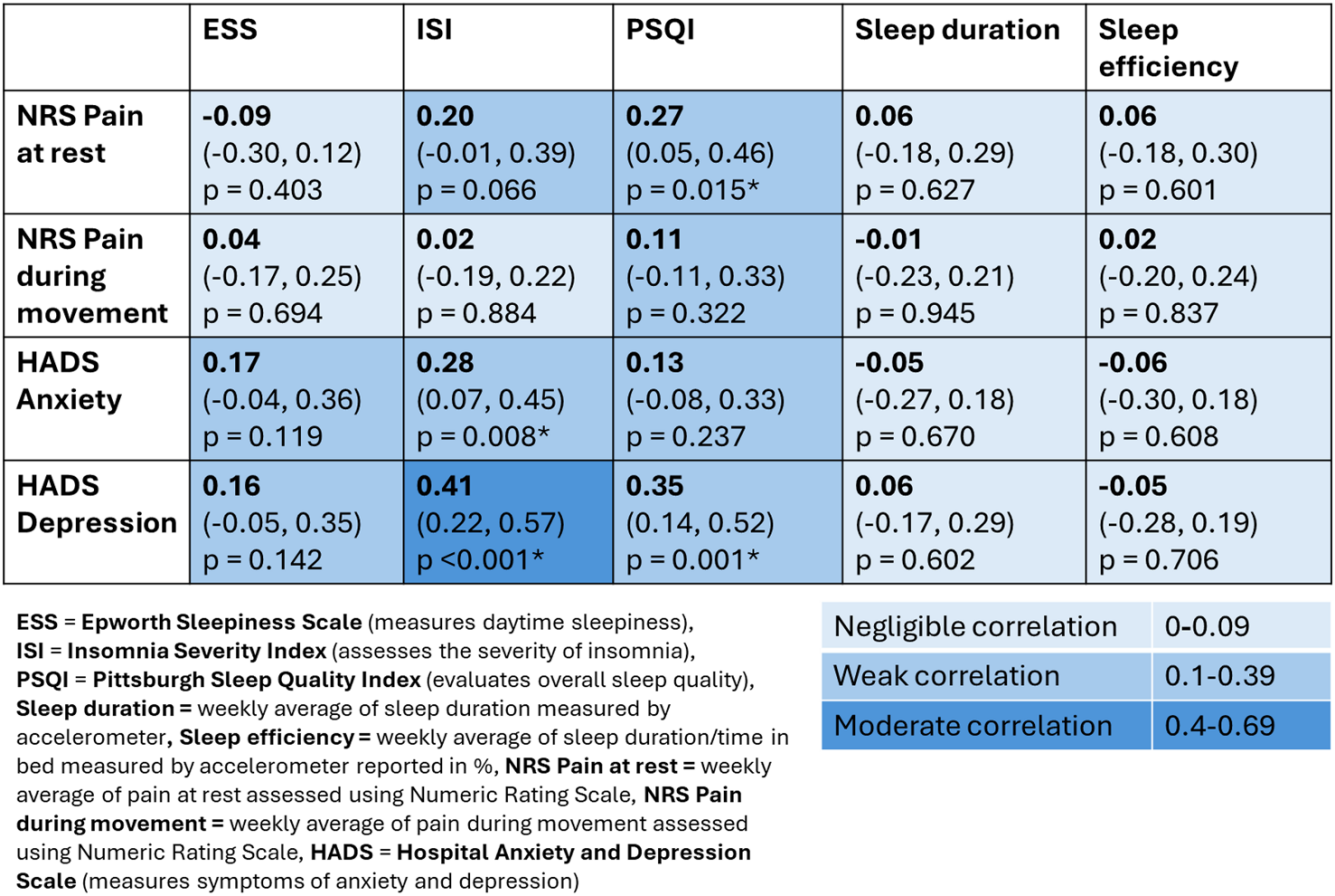
Spearman's correlations between sleep–wake disturbances, pain, and symptoms of depression and anxiety. *Significant at the 0.05‐level.

There were weak correlations between ISI scores and symptoms of anxiety according to HADS, between PSQI scores and pain at rest according to the NRS, and between PSQI scores and symptoms of depression according to HADS. A moderate correlation was found between ISI scores and depression (*r*_s_ = 0.41, 95% CI, 0.22–0.57) (Figure 5).

## 4 Discussion

This study highlights the high prevalence of symptoms of sleep–wake disturbances in patients with chronic pain. In this cohort, we were able to demonstrate a substantial overall burden of impaired sleep, encompassing multiple dimensions such as sleep quality, duration, and daytime sleepiness using reliable and validated questionnaires, detailed sleep diaries, as well as objective accelerometry measurements. From a clinical perspective, this underscores the importance of systematically assessing sleep in this patient group, as a broad range of symptoms of sleep–wake disturbances may otherwise remain unrecognised and untreated. Importantly, the present study was not designed to establish formal clinical diagnoses of specific sleep disorders, such as insomnia, RLS, or OSA, which require comprehensive clinical assessment. Moreover, there is considerable overlap between different sleep–wake disturbances, and patients may report symptoms consistent with more than one condition. For example, symptoms of insomnia may coexist with symptoms associated with OSA and daytime sleepiness. This overlap makes it challenging, and potentially of limited clinical relevance, to quantify the number of specific sleep disorders within this population based on the available data. Therefore, our findings should be interpreted as reflecting the presence of symptoms of sleep–wake disturbances rather than the prevalence of distinct diagnostic entities in this population.

Similar prevalence of insomnia symptoms, as found in our study, have been reported in other studies of patients with chronic pain (Gerdle et al. 2023). In a recent meta‐analysis of studies conducted in the general population, van Straten et al. reported a pooled prevalence of 7.5% (95% CI, 4.8%–11.4%) for clinical insomnia when using an ISI cut‐off score of ≥ 15, whereas in our sample, 67% exceeded this threshold (van Straten et al. 2025). This finding exemplifies the high burden of insomnia in our cohort. Our collective findings underscore the importance of implementing sleep assessment in patients with chronic pain. Complementary measurements using accelerometry, self‐assessment questionnaires, and sleep diaries provided all useful information, even though sleep duration from the diaries overestimated sleep time by a little over an hour compared with accelerometry in our study. Nevertheless, the mean sleep duration in the population remained below the recommended 7 h. An overestimation by 30–60 min when using self‐report compared with actigraphy has also been described elsewhere (Chaput 2025).

Previous research has consistently reported associations between sleep duration, health, and all‐cause mortality. A systematic review and meta‐analysis by Cappuccio et al. identified a U‐shaped relationship, indicating that both short (< 5 h) and long (> 9 h) sleep durations were associated with an increased risk of mortality (Cappuccio et al. 2010). Sleep duration between 7 and 9 h has been recommended by the American Academy of Sleep Medicine (Watson et al. 2015). As further support for these findings, Jin et al. reported a similar U‐shaped association, with 7 h per night associated with the lowest risk of mortality (Jin et al. 2022). In the present study, 40% of patients slept < 5 h per night, placing them in an increased risk category. Yet, the combination of disturbed sleep time and sleep efficiency, as seen in our study, seems to present even greater concerns from a health perspective. In a UK Biobank study encompassing over 90,000 participants and using accelerometer‐based measurements, both sleep duration and sleep efficiency were independently associated with mortality, and the combination of short (< 6 h) or long (> 8 h) sleep duration with low sleep efficiency (≤ 71.9%) further increased the risk (Liang et al. 2023).

Along with sleep disturbances, we observed low levels of physical activity assessed with accelerometry among the study participants, a factor also known to adversely affect health outcomes. Time spent in MVPA was, on average, 41 min/week, compared to the WHO recommendations of 150–300 min/week. In a cross‐sectional study from an unselected population (*n* = 2649), higher levels of physical activity were associated with improved sleep efficiency, but not sleep duration, insomnia, or daytime sleepiness, after adjusting for BMI (Gubelmann et al. 2018). We reached consistent findings in this cohort with chronic pain, although only a weak correlation was seen between physical activity and sleep efficiency. This difference may partly be explained by the low total level of physical activity measured by accelerometry in our cohort. Poor sleep quality, as well as high pain intensity, could have been barriers for the patients to engage in physical activity, which can create a vicious circle that exacerbates inactivity and fatigue. These barriers to physical activity can also negatively affect the process of recovery from pain and are important to assess clinically.

Regarding measuring physical activity, accelerometer‐based estimates did not correspond well with self‐reported data, and self‐reported physical activity was not associated with any sleep outcomes. Notably, only accelerometer‐measured physical activity was associated with accelerometer‐derived sleep efficiency. This discrepancy may reflect differences in what the two methods capture. Self‐reported measures primarily assess intentional physical activity and may be influenced by recall bias and overestimation, whereas accelerometry provides an objective measure of total daily movement, including light activity and sedentary behaviour. This distinction may be particularly relevant for sleep, which may be influenced by overall 24‐h activity patterns rather than isolated exercise sessions. Importantly, the aim of the study was not to directly compare methods for assessment, but to use complementary approaches. Together, these findings suggest that accelerometer‐based measures may be more sensitive for detecting associations between physical activity and sleep.

A review by Husak and Bare found that patients experiencing both chronic pain and sleep disturbances reported higher pain intensity, longer pain duration, greater levels of disability, and lower levels of physical activity compared to those without sleep disturbances (Husak and Bair 2020). Interestingly, our study could not confirm these correlations. In terms of pain and physical activity, we did not find differences between participants with moderate or severe insomnia compared with those with no or mild insomnia. However, those with moderate or severe insomnia did report more symptoms of depression. A possible reason for the absence of such associations is that our cohort may include individuals with more severe complications, who more commonly report poor sleep quality, high pain intensity, and low levels of physical activity. This reflects the population typically seen at specialist pain clinics in Sweden reporting to the SQRP (Lind et al. 2021).

Lastly, the current results did not find differences between participants who used opioids and non‐users, except for a higher risk score according to the STOP‐Bang questionnaire for OSA among opioid users. In this context, it may be useful to actively screen individuals with chronic pain for OSA as well as RLS. These conditions were prevalent in this cohort and may arise as side effects of pharmacological treatment, particularly in those treated with opioids.

This present study has several strengths. First, the use of validated questionnaires in combination with accelerometer data and sleep diaries provides a multidimensional perspective on sleep disturbances experienced by individuals with chronic pain. Second, the sample size (*n* = 100) is relatively large, considering that this was a clinical study involving patients with complex and severe pain referred to a tertiary pain clinic. Participation in the study may have been challenging for many, considering the overall burden of the condition.

One limitation of the study is that a substantial number of eligible patients declined participation, which may have led to selection bias. Nevertheless, the patient characteristics (demographic as well as questionnaire data) in our study are comparable with those of patients in the SQRP, which supports the generalisability of the results. Another limitation of this study was the relatively high proportion of missing data, particularly regarding reported opioid doses. This limited our ability to fully address one of our research questions concerning the impact of opioids on sleep. It is also possible that multiple factors, including sleep disturbances, pain severity, and reduced physical activity, may have influenced the participants' ability to complete the questionnaires, as well as their subjective perception of sleep. Finally, the estimation of OSA frequency was based on the STOP‐Bang questionnaire, which systematically underestimates sleep apnea in females. As this cohort was predominantly female, the true influence of sleep apnea on pain, sleep, and activity may be underestimated, and further studies using objective measures of respiration during sleep in this patient group are warranted.

### 4.1 Clinical Implications

Sleep–wake disorders are challenging to treat and often underestimated in patients with chronic pain, which might further contribute to impaired pain modulation. The heterogeneity of sleep–wake disorders underscores the need for thorough individual assessment as well as tailored treatment of sleep impairment. Anxiety, depression, and physical inactivity are also important contributing factors to sleep–wake disturbances and should be investigated.

## 5 Conclusion

Symptoms of a variety of sleep–wake disturbances are present in patients with chronic pain, and none of the participants in this study reached the recommendations for > 85% sleep efficiency indicating stable sleep. This underscores the importance of assessing sleep to optimise treatment and rehabilitation of chronic pain. A weak correlation was identified between sleep efficiency and physical activity, and a moderate correlation between symptoms of insomnia and symptoms of depression, indicating the importance of also assessing mental health and physical inactivity in individuals with chronic pain. There is a need to further map the impact of sleep–wake disturbances in chronic pain.

## Author Contributions

P.A., M.O., E.V., J.H., L.G., M.B., and D.A. conceived and designed the study. P.A., E.V., and M.O. collected the data. E.V., A.P., and M.O. performed the analyses. All authors interpreted the data, contributed to drafting or critically revising the manuscript, and approved the final version.

## Funding

The study was financed by grants from the Swedish governmental funding of clinical research (ALF) (Grant ALFGBG‐965210 and ALFGBG‐1005118), and the Gothenburg Society of Medicine (Grant GLS‐961176).

## Conflicts of Interest

L.G. reports lecturing activities for Resmed, Philips, Itamar, Astra Zeneca, and Lundbeck. L.G. is member of the advisory board for the medical device company ONERA BV and he is shareholder of a company with a licensed patent on pharmacological treatment in sleep apnea. P.A. is president of the Swedish Pain Society and a member of the steering committee for Societal Impact of Pain Sweden. No other conflicts of interest are reported.

## Supporting information


**Table S1:** Sleep, pain, physical activity, and symptoms of anxiety and depression in participants using opioids vs. not using opioids.
