# Supplementary material for: Sleep–Wake Disturbances in Patients With Chronic Pain—Associations With Physical Activity Levels
Source: Eur J Pain. 2026 Jul 6;30(6):e70330. doi: 10.1002/ejp.70330 (PMC13338585; doi:10.1002/ejp.70330)
Supplement: Supplementary file 1 — Table S1: Sleep, pain, physical activity, and symptoms of anxiety and depression in participants using opioids vs. not using opioids. [file EJP-30-0-s001.docx]

**Supplementary Table 1.** Sleep, pain, physical activity, and symptoms of anxiety and depression in participants using opioids vs not using opioids.

|  | **Using opioids**  **(n = 33)** | **Not using opioids**  **(n = 67)** | **Mean difference (95% CI)^a^** |
| --- | --- | --- | --- |
| Age (years) | 48 (12) | 47 (14) |  |
| Gender, women/men, n | 21/12 | 41/25 |  |
| BMI (kg/m²)  Morphine equivalents (ME)^b^ | 27.5 (6.6)  43.2 (48.7) | 28.2 (6.5)  0.0 (0.0) |  |
|  |  |  |  |
| **SLEEP** |  |  |  |
| *Accelerometer* |  |  |  |
| Sleep duration (hh:min) | 5:10 (1:55) | 5:41 (1:42) | -31.4 (-91.2 – 28.4)^c^ |
| Sleep efficiency (%, *sleep duration/total time in bed)*) | 62 (16) | 65 (12) | -2.0 (-9.3 – 5.3) |
| *Diary, 7-day average*  Estimated sleep duration (hh:min) | 6:30 (1:27) | 6:33 (1:10) | -3.5 (-43.8 – 36.7)^c^ |
| *ISI score (0-28p)* | 16.4 (7.8) | 17.0 (6.5) | -0.4 (-3.8 – 3.0) |
|  |  |  |  |
| *ESS score (0-24p)* | 8.8 (5.7) | 8.3 (5.3) | 1.1 (-1.5 – 3.7) |
|  |  |  |  |
| *PSQI score (0-21p)* | 11.5 (4.8) | 12.2 (3.6) | -0.5 (-2.5 – 1.4) |
|  |  |  |  |
| *RLS score (0-4p)* | 1.8 (1.6) | 1.7 (1.6) | 0.2 (-0.6 – 1.0) |
|  |  |  |  |
| *STOP-Bang score (0-8p)* | 3.0 (2.0) | 2.5 (1.7) | 0.7 (0.2 – 1.2)** |
| **PAIN** |  |  |  |
| *NRS, (0-10), 7-day average rest* | 5.7 (2.1) | 6.0 (1.8) | 0.2 (-1.1 – 0.8) |
| *NRS (0-10), 7-day average motion* | 6.1 (2.2) | 6.5 (1.9) | -0.4 (-1.4 – 0.7) |
|  |  |  |  |
| **PHYSICAL ACTIVITY** |  |  |  |
| *Accelerometer,*  *MVPA min/week* | 41 (47) | 40 (49) | -0.5 (-24.5 – 23.6) |
| *Self-reported*  *MVPA min/week* | 252 (177) | 194 (152) | 63 (-12.1 – 137.5) |
|  |  |  |  |
| **ANXIETY AND DEPRESSION** |  |  |  |
| *HADS Anxiety, (0-21p)* | 7.5 (5.2) | 9.4 (5.0) | -1.3 (-3.7 – 1.2) |
| *HADS Depression, (0-21p)* | 8.5 (4.6) | 9.4 (4.8) | -0.4 (-2.7 – 1.9) |

Data are reported as mean (SD). ^a^Adjusted for BMI, gender, and age. ^b^Ten participants did not report the dose of opioids. ^c^Mean difference reported in minutes. **p < 0.01 **ISI** = **Insomnia Severity Index** (assesses the severity of insomnia), **ESS** = **Epworth Sleepiness Scale** (measures daytime sleepiness), **PSQI** = **Pittsburgh Sleep Quality Index** (evaluates overall sleep quality), **RLS** = **Restless Legs Syndrome** (a neurological condition causing an urge to move the legs), **MVPA** = **Moderate-to-Vigorous Physical Activity** (measures level of physical activity), **NRS** = **Numeric Rating Scale** (used for pain intensity assessment), **HADS** = **Hospital Anxiety and Depression Scale** (measures symptoms of anxiety and depression)
